# Supplementary material for: The Furvela tent-trap Mk 1.1 for the collection of outdoor biting mosquitoes
Source: PeerJ. 2017 Nov 15;5:e3848. doi: 10.7717/peerj.3848 (PMC5694212; doi:10.7717/peerj.3848)
Supplement: Supplemental Information 1 [file peerj-05-3848-s001.docx]

The Furvela Tent Trap mk1.1

J.D. Charlwood.

#
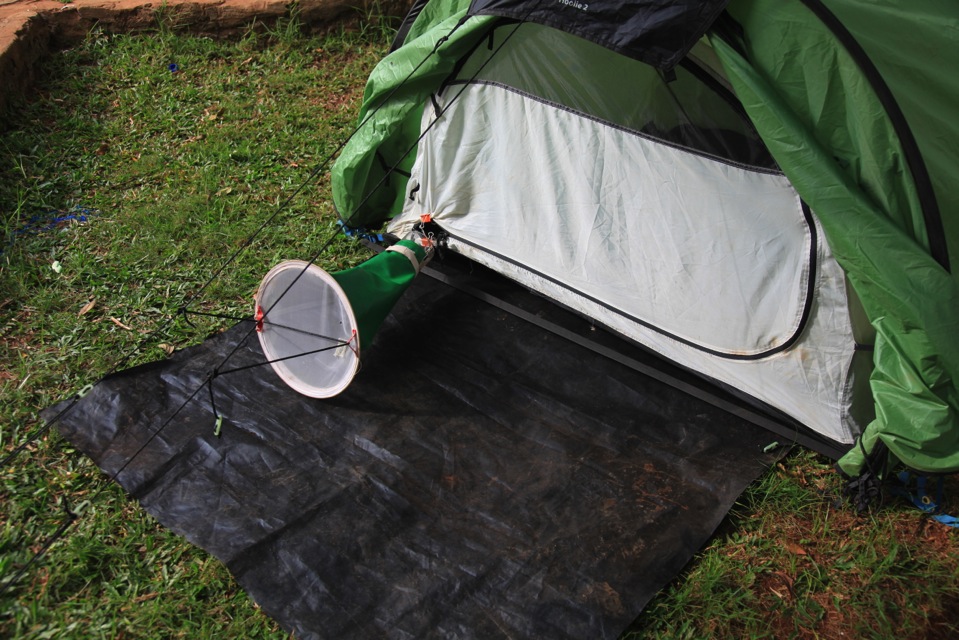


# Purpose and Principle

The purpose of this document is to provide step-by-step instructions on how to assemble a Furvela tent-trap to conduct the collection of outdoor biting mosquitoes.

Mosquitoes, attracted to the odour plume emanating from an opening at the front or side of the tent (and drawn out by the trap’s fan), are sucked into the trap when they approach it. Even in areas with very high densities of mosquitoes none will enter the tent so that exposure to bites does not occur.

Equipment/Material needed:

The trap uses any commercial tent that has a door at the sleepers’ head or side that is not covered with an external flysheet. Such tents all have one or two zippered openings that act as a door. Tents like these weigh only a few kilos and so can easily be backpacked to remote areas for mosquito collection if required.

**Single-door tents** that have been used include: Arenas 2 from Quecha (Decathlon www.decathlon.co.uk £20 (€28 or 32$)*),* Camp Dome 200 and 400 from Campmaster [www.**campmaster**.co.za](http://www.campmaster.co.za): the Vaude Ultra light 2 www.**vaude**.com and the *Hoolie Wildcountry 2*

**Double door tents** that have been used include the Glen Orchy Highlander2 evaq8.co.uk, the Quecha 2 door for £48 (€68 or 75$) www.decathlon.co.uk and the Nemo Losi 3 person tent [www.nemoequipment.com](http://www.nemoequipment.com)

A ‘**footprint**’ for the tent. This can be purchased from the tent supplier but a piece of plastic sheet works just as well.

One **CDC light-trap model 512** without the light, lid or grid.

A **cone type collection bag** (available from John Hock – part 1.42 price 18$), johnwhock.com/products

Two medium or small **bulldog’ or ‘binder’ clips’** [www.**staples**.com](http://www.staples.com) or [www.officedepot.com](http://www.officedepot.com)

**Needle and thread**

**16 – 18 gauge wire** Two pieces 6-7 cm long (≈ 1mm in diameter or ≈)

A 6V sealed lead acid **rechargeable battery** (4.5 Ah and above)

Battery **charger** for above.

**Camping-mat** and **sleeping bag**

### Preparation


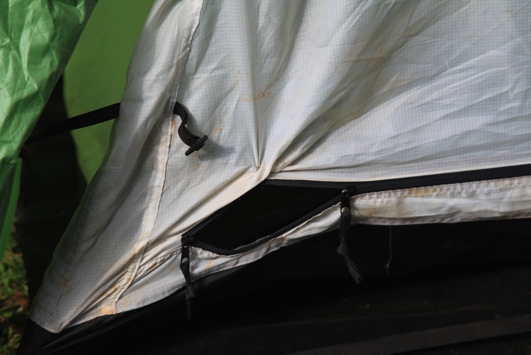


Sew the end of the zip back onto itself to keep the *do*or of the tent open at one end of the zip.. The opening should be approximately the same size as the diameter of a CDC light-trap.

Remove, the lid, light and from a standard CDC miniature light-trap.

Make two holes, circa 2mm in diameter close to the top of the Perspex of the trap.


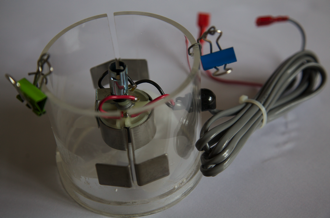


Holes can be made either with a hot wire or a small drill.

Pass a small (6-7 cm) length of stiff, but malleable, wire (≈ 1mm in diameter or ≈ 16 – 18 gauge wire,) through each hole

Attached the wires to one handle of a medium or small ‘bulldog’ clip. The wire can easily be used to make the holes in the Perspex. Heat it until it is red hot in a flame (from, say, a gas cooker) and immediately, gently push the wire through the Perspex to make the hole.


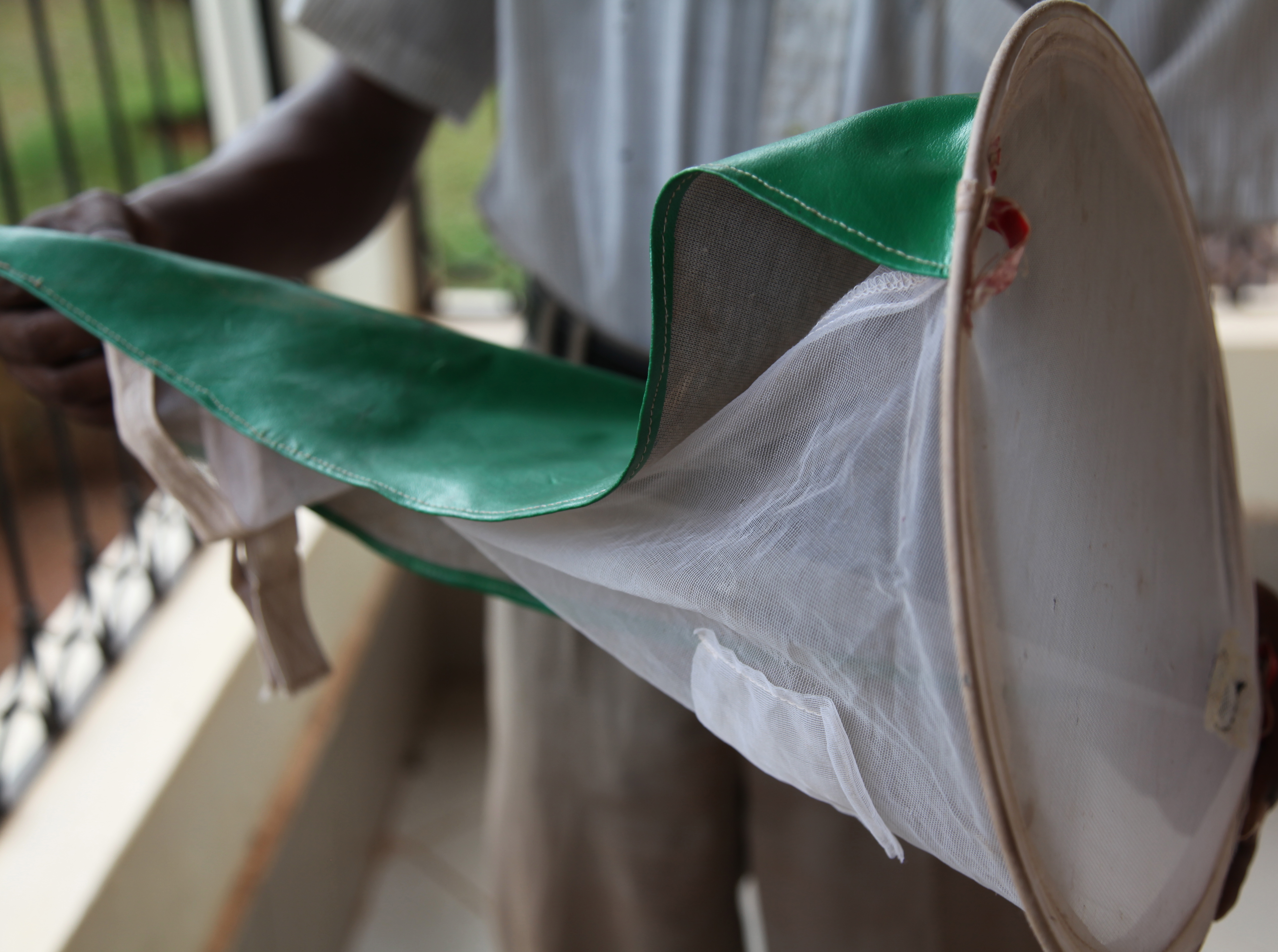


Sew two eyelets sewn at the bottom of the collection bag and sew a waterproof cover on to the top half of the bag.

# Procedure

## The tent

1. If performing paired indoor sampling, attempt select an area located approximately 10m the dwelling that is relatively flat and devoid of rocks. Install the tent after first laying out the footprint. The footprint should extend circa 60cm in front of the tent door. This makes both entering and leaving the tent more comfortable and also reduces the likelihood that ants or other predators might find their way into the trap.
2. Some tents (such as the Wild Country Hoolie 2) already come with guy ropes in front of the door. If they do not two ropes need to be attached. This is generally not a major difficulty.

## Installation


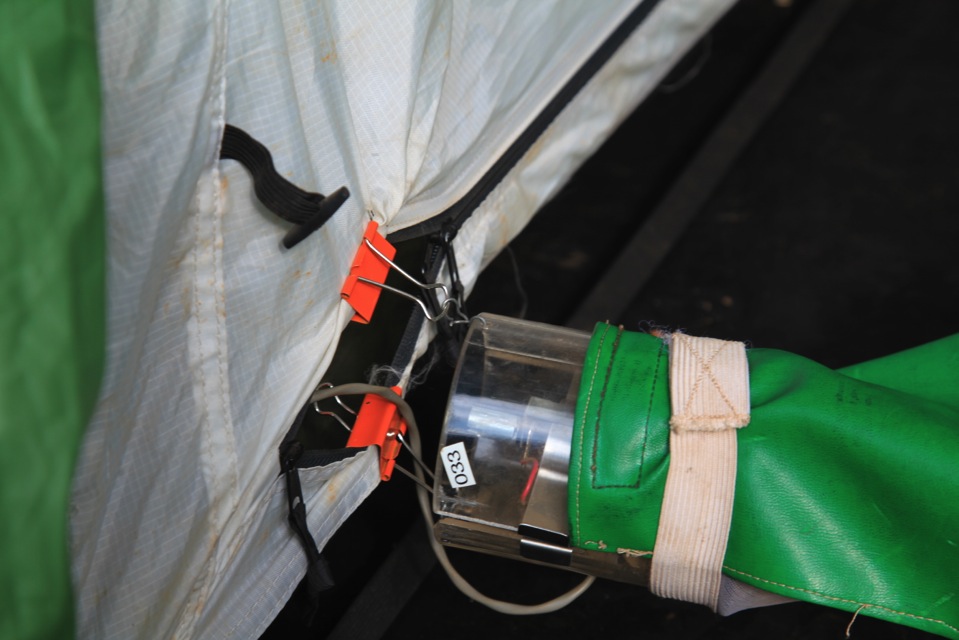
Pass the CDC trap wire through the opening so that the occupant of the tent can attach the battery to the trap once they are inside.

Attach the body of the CDC trap to the opening of the tent using the bulldog clips so that it is suspended horizontally, circa 3cm in front of the opening of the tent door.


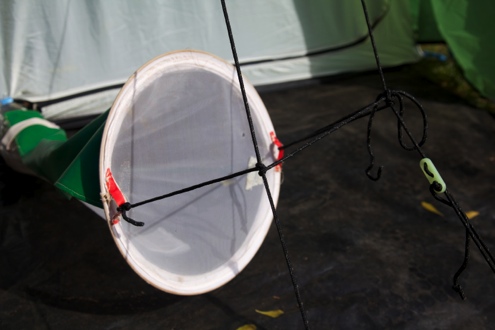
Attach the collection bag to the trap and to the guy ropes in front of the door. A string is threaded through the eyelets and attached to the guy ropes in front of the door, using a simple over and under knot like the ‘cow hitch’ to prevent the bag sliding down the rope (www.netknots.com). The string can also be attached to a separate pole. The tension on the collection bag keeps the trap horizontal. Thus, the only attachment by which possible predators or scavengers of the collected mosquitoes can enter the trap are via the two clips that attach the trap to the tent, or the string that is used to attach the bag to the guy ropes.


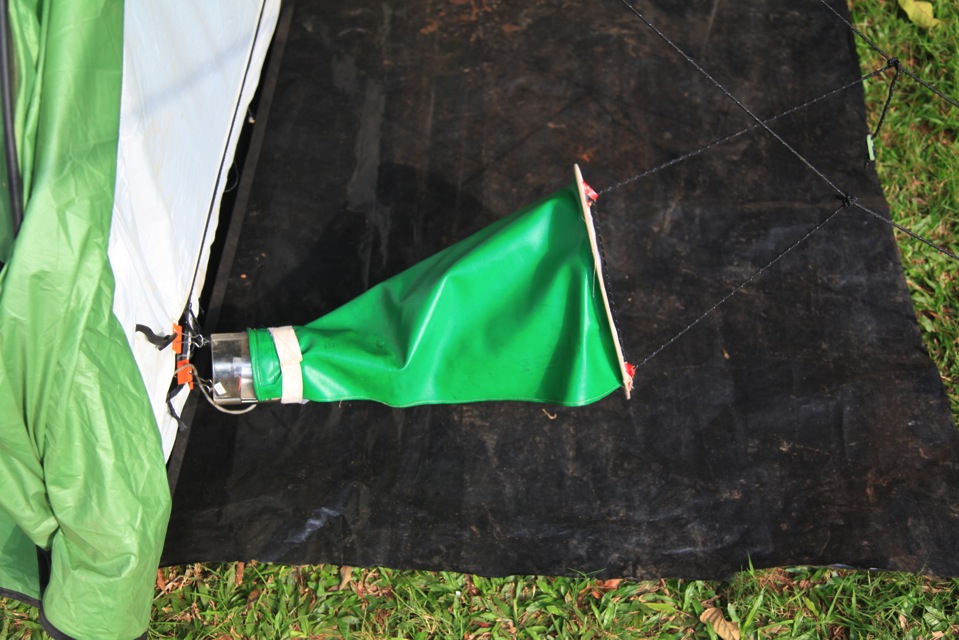


### Installation of the trap

### Enhancing survival rates

If cotton wool soaked in a weak glucose solution (or just water) are added to the pockets provided on the sides of the collection bag then survival, at least among the malaria vectors, is high. In Ghana 2119 of 3133 (6(%) *An. gambiae* had survived until the morning and more recently 1744 of 3089 (56%) *An. gambiae* s.l. survived until the morning (compared to fewer than 3% that survived in light trap collections). Recently killed mosquitoes are easier to dissect for age determination although there is a bias towards recently emerged insects among live insects (Charlwood et al., 2011, Charlwood & Tomás unpublished data). This may be because these insects are more likely to sugar feed (and so survive) rather than any intrinsic advantage (Charlwood et al.,1998), and so in order to obtain unbiased estimates of survival dead insects should also be examined if possible.

Information on the collection (such as date, location, time interval and the name of the sleeper) can be put into the collection bag before it is attached to the trap or placed in the pocket of the bag.

### Collection

The standard time for the host to enter the trap depends on the mosquito that one wants to collect. For anophelines and other night biting mosquitoes the time to enter the trap is shortly after, or at, sunset. In order to estimate exposure of an ‘average’ person the collection bag can be changed at the time that people usually go inside their houses at night, which can be ascertained by observation. In order to determine biting activity during the night the collection bags can also be changed at regular intervals.

The collector enters in the tent trap with mat, sleeping bag and the battery for the trap at sunset. As DC motors reverse their direction of rotation with voltage polarity changes, care needs to be taken that the red (+) battery lead is attached to the positive terminal of the battery and the black (-) lead is attached to the negative terminal of the battery.

Sleep and/or change collection bags at set times of the night.

In the morning before disconnecting the battery from the trap, remove and tie the sleeve of the collection bag to avoid mosquitoes escaping.

### Battery charging

Charge the battery after a single nights use to avoid complete discharge Since the trap does not use a light the energy requirements are only approximately half those of a CDC light-trap (170 mAmp/hr compared to 320mAmps/hr for the trap with a bulb). Thus, a 6V 4.5 Ah battery, weighing just 400 grams can power the trap.

### Other information


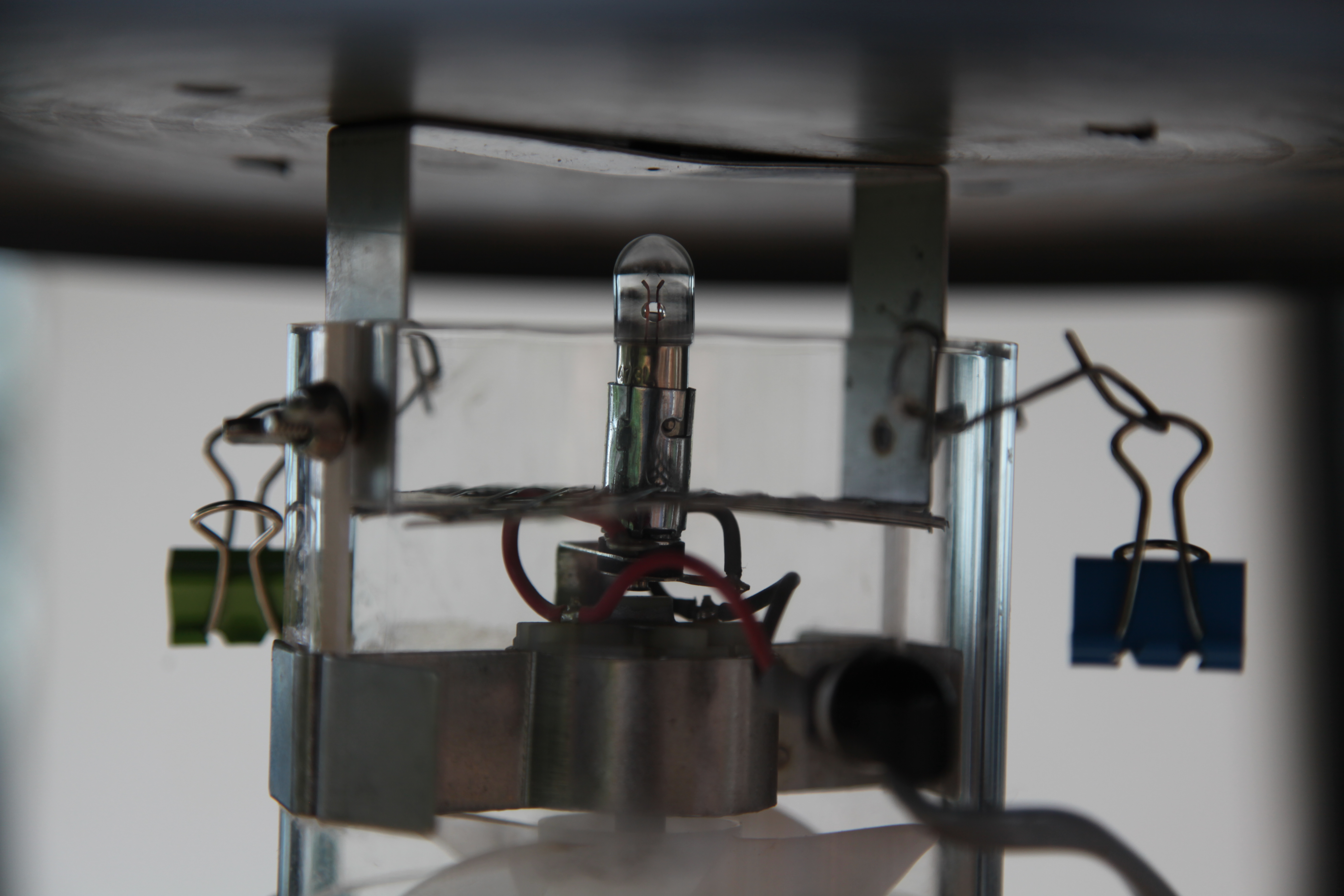


When not used as a tent-trap the CDC trap

can easily be reconverted to the standard

light-trap without the need to remove the

clips since they hang outside of the trap body.

The tent can also, of course, be used as a normal tent.

To see a video of trap preparation and installation go [www.youtube](http://www.youtube). MOZDAN.
